# The mitochondrial genome of *Rhaphium baihuashanum* (Diptera: Dolichopodidae)

**DOI:** 10.1080/23802359.2018.1507644

**Published:** 2018-10-29

**Authors:** Qilemoge Qilemoge, Junhua Zhang, Ding Yang

**Affiliations:** a College of Plant Protection, China Agricultural University, Beijing, China;; b Chinese Academy of Inspection and Quarantine, Institute of Plant Quarantine, Beijing, China

**Keywords:** Mitochondrial genome, rhaphiinae, phylogenetics

## Abstract

The long-legged fly *Rhaphium baihuashanum* Yang belongs to the subfamily Rhaphiinae of Dolichopodidae. The mitogenome of *R. baihuashanum* was sequenced, the first representative of the mitogenome of the subfamily. The mitogenome is 15,173 bp totally, consisting of 13 protein-coding genes, two rRNAs and 22 transfer RNAs. All genes have the similar locations and strands with that of other published species of Dolichopodidae. The nucleotide composition biases toward A and T, which together made up 74.1％ of the entirety. Bayesian inference analysis strongly supported the monophyly of Dolichopodidae. It suggested that Rhaphiinae is the sister group to the clade of ((Hydrophorinae + Neurigoninae) + (Sciapodinae + Dolichopodinae)).

Rhaphiinae is a large subfamily of the family Dolichopodidae, and the genus *Rhaphium* is the largest genus of the subfamily Rhaphiinae with 199 species known from the world (Yang et al. 2006; 2011; Grichanov 2017). Primarily distributed in the Holarctic realm, Oriental realm and Ethiopian realm, species of *Rhaphium* are usually found in the habitats near freshwater on the stones and plants in or near the streams.

The adult specimens of *R. baihuashanum* used for this study were collected from Hohhot (40°81′501″N, 111°33′991″E, 1300 m) of Inner Mongolia in China in 2017. The specimens of *R. baihuashanum* were deposited in the Entomological Museum of China Agricultural University (CAU). The total genomic DNA was extracted from the whole body (except head) of the specimen using the QIAamp DNA Blood Mini Kit (Qiagen, Germany) and stored at −20 °C until needed. The nearly complete mitogenome of *R. baihuashanum* is 15,173bp. It encoded 13 PCGs, 22 tRNA genes, 2 rRNA genes and the control region could not be sequenced entirely in this study, and were similar with related reports before (Kang et al. 2014; Li et al. 2016; Wang, Ding, et al. 2016; Wang, Wang, et al. 2016; Wang, Liu, et al. 2016; Li et al. 2017; Zhou et al. 2017; Qilemoge et al. 2018). The nucleotide composition of the mitogenome was biased toward A and T, with 74.1% of A + T content (A = 39.8%, T = 34.3%, C = 15.7%, G = 10.2%). The A + T content of PCGs, tRNAs, and rRNAs is 72.1%, 75.6%, and 78.9% respectively. The total length of all 13 PCGs of *R. baihuashanum* is 11,084 bp. All PCGs in *R. baihuashanum* utilize the conventional translational start codons for invertebrate mtDNA. For example, five PCGs (*COII*, *COIII*, *ATP6*, *ND4* and *CYTB*) initiated with ATG codons, three PCGs (*ND2*, *ND3* and *ND4L*) initiated ATT codons, three PCGs (*ND1*, *ND5* and *ND6*) initiated ATA codons, *ATP8* initiated with ATC as a start codon, *COI* initiated with TCG as a start codon. Eleven PCGs used the typical termination codons TAA and two PCGs (*CYTB*, *ND3*) used TAG in *R. baihuashanum*.

Phylogenetic analysis was performed based on the nucleotide sequences of 13 PCGs and 16S (rRNA) from 13 Diptera species. Bayesian (BI) analysis (Figure 1) showed that monophyletic Empidoidea was assigned to be the sister group to the clade of Xylophagidae and Asilidae. For the phylogeny of Empidoidea, monophyletic Empididae was assigned to the sister to monophyletic Dolichopodidae. For the phylogeny of Dolichopodidae, Rhaphiinae was assigned to be the sister of the clade of Neurigoninae, Hydrophorinae, Sciapodinae and Dolichopodinae. The phylogenetic relationship within Empidoidea is very clear: Empididae + (Rhaphiinae + ((Hydrophorinae + Neurigoninae) + (Sciapodinae + Dolichopodinae))). The position of Rhaphiinae was also supported by the morphological study (Yang et al. 2006; 2011). The mitogenome of *R. baihuashanum* could provide the important information for the further studies of Dolichopodidae phylogeny.

**Figure 1.**
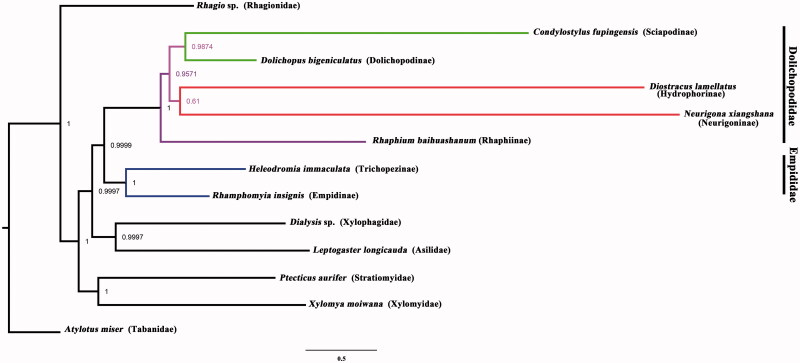
Bayesian phylogenetic tree of 13 Diptera species. The posterior probabilities are labeled at each node.
